# Ankle Arthrodesis Revisited: A Systematic Review of Techniques, Outcomes, and Complications

**DOI:** 10.7759/cureus.86836

**Published:** 2025-06-27

**Authors:** Muhammad Y Raufi

**Affiliations:** 1 Trauma and Orthopaedics, Leeds Teaching Hospitals NHS Trust, Leeds, GBR

**Keywords:** ankle and foot, ankle arthrodesis, ankle fusion arthroplasty, ankle osteoarthritis, orthopaedics and traumatology

## Abstract

Introduction: Ankle arthrodesis is a widely accepted treatment for managing end-stage ankle arthritis and various other conditions, including post-traumatic, inflammatory, congenital, and neurogenic deformities. Several surgical techniques for ankle arthrodesis have been developed, such as open ankle fusion, arthroscopic ankle fusion, and mini-open ankle arthrodesis. The purpose of this study was to explore the range of surgical approaches and techniques used in ankle arthrodesis, with particular emphasis on comparing their outcomes in terms of complications, hospital stay duration, pain relief, and functional improvement.

Methodology: This systematic review was conducted following the Preferred Reporting Items for Systematic Reviews and Meta-Analyses (PRISMA) guidelines. The review included comparative studies, including randomized controlled trials, non-randomized controlled trials, and observational studies, that assessed patients undergoing ankle arthrodesis.

Results: The study analyzed various surgical techniques, including the anterior versus transfibular approach, Ilizarov versus internal fixation, and open versus arthroscopic techniques. The results indicated that the visual analogue scale (VAS) scores did not show a statistically significant difference between the anterior and transfibular approaches or between the Ilizarov and internal stability methods. However, the length of hospital stay was statistically significant (p = 0.05) when comparing the arthroscopic technique (mean 2.5 days) to the open technique (mean 3.7 days). The comparison between the Ilizarov and internal stability techniques did not show a significant difference in length of stay. A statistically significant difference in postoperative alignment was observed between the anterior and transfibular approaches, with the transfibular approach resulting in an average valgus alignment of 2.4°.

Different studies employed various scoring systems to evaluate clinical outcomes, including the American Orthopaedic Foot & Ankle Society Score (AOFAS), ankle osteoarthritis scale (AOS), foot and ankle ability measure (FAAM), and short form (36) (SF-36) scores. No significant differences were found between the techniques, except for the comparison between open and arthroscopic approaches. The arthroscopic technique demonstrated significantly better outcomes in terms of the AOS score (p = 0.05) and the physical component of the SF-36 (p = 0.01).

Conclusion: Further research is necessary in this field, and it is essential for surgeons to reach a consensus on the most effective techniques for ankle arthrodesis, rather than employing a variety of approaches.

## Introduction and background

Ankle arthrodesis is a commonly accepted treatment for end-stage ankle arthritis and a range of other common issues, including post-traumatic, inflammatory, congenital, and neurogenic deformities, which lead to pain and restricted movement in the ankle joint [1]. In a multicentre randomised controlled trial, Goldberg et al. in 2015 compared the outcomes of total ankle replacement vs. arthrodesis for end-stage ankle osteoarthritis. The conclusion was that both total ankle replacement and arthrodesis improved the Manchester-Oxford Foot Questionnaire walking/standing (MOXFQ-W/S) score and had similar clinical scores and adverse events. It was found that ankle arthrodesis was superior to total ankle replacement when it came to wound-related infections and nerve injuries [2]. Therefore, looking into the approaches and techniques used for ankle arthrodesis is important.

At least 40 different techniques for ankle arthrodesis have been developed, such as open ankle fusion, arthroscopic ankle fusion, and mini-open ankle arthrodesis [3]. Open ankle arthrodesis involves a wide range of surgical techniques, including variations in approach, fixation methods, and postoperative care. Even within these techniques, there are more specific options, such as the lateral approach, which could involve a high or low distal fibular resection; a sagittal osteotomy with reattachment of part of the fibula, or keeping the fibula fully intact [4]. This study aimed to investigate various surgical approaches and techniques used in ankle arthrodesis, with a focus on comparing their outcomes in terms of complications, length of hospital stays, pain relief, and functional outcome.

## Review

Methodology

**Figure 1:**
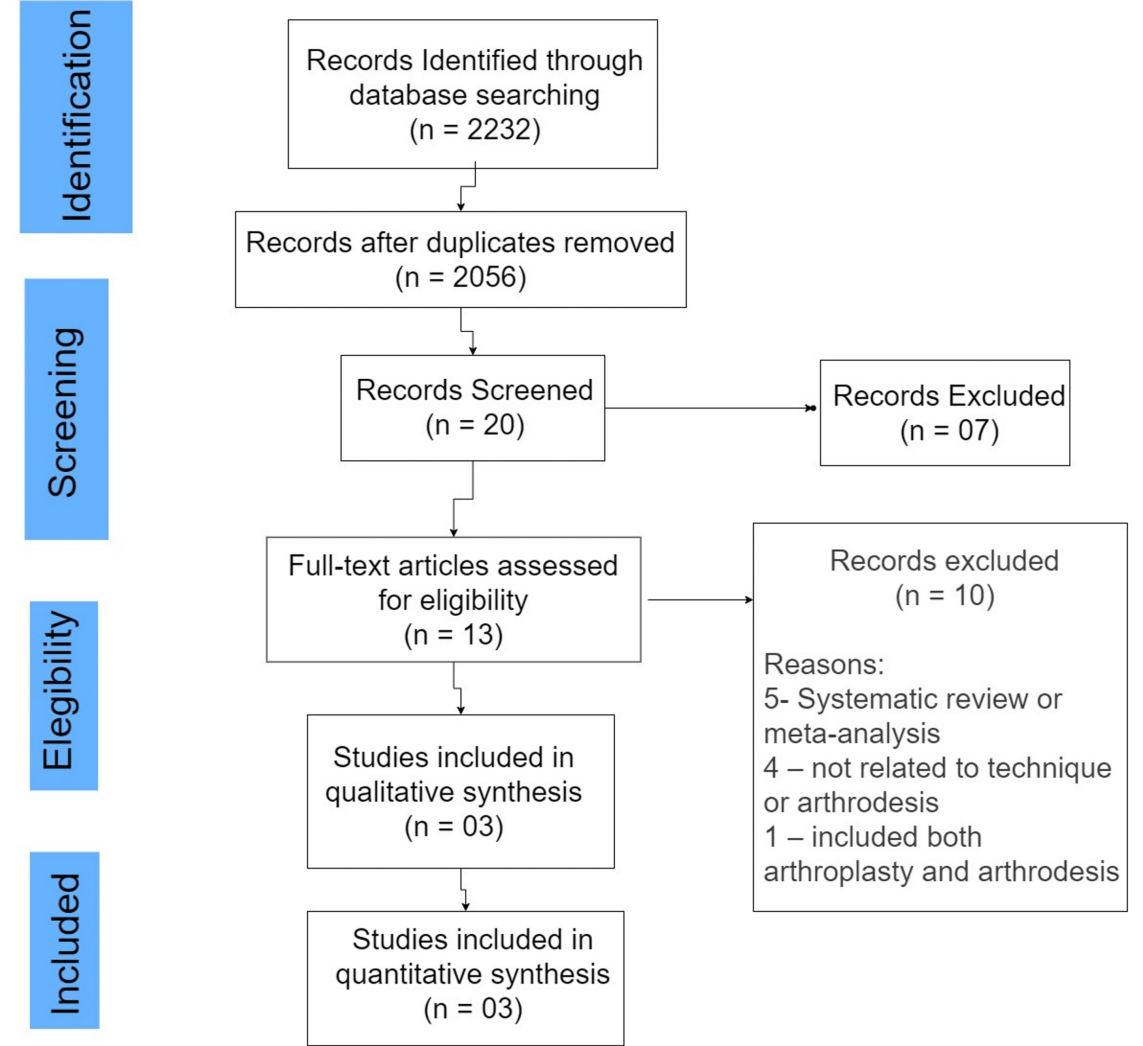
PRISMA flow chart depicting article screening and selection for assessment of different techniques of ankle arthrodesis PRISMA: Preferred Reporting Items for Systematic Reviews and Meta-Analyses

This systematic review was conducted per the Preferred Reporting Items for Systematic Reviews and Meta-Analyses (Figure 1) [5].

Eligibility Criteria

All comparative studies, including randomized as well as nonrandomized controlled trials and observational studies comparing those who underwent ankle arthrodesis, were included. There was no restriction for eligibility irrespective of age, sex, or comorbidity status. Articles older than five years, systematic reviews, and meta-analyses on similar topics were excluded from the review process, as well as articles not reported in English.

Literature Search Strategy

The author independently searched the electronic databases of MEDLINE, Embase, CINAHL, PUBMED, Google Scholar, and the Cochrane Central Register of Controlled Trials (CENTRAL). The last search was conducted on 19th February 2024. Medical subject headings (MeSH) terms including arthrodesis/methods, osteoarthritis/surgery, ankle Joint, and surgery with all terms combined using adjuncts of “and” as well as “or" were used.

Selection of Studies

Initially, there were 75,943 results. Then the author used the eligibility criteria of articles being from the past five years and only clinical trials, meta-analyses, systematic reviews, and randomized controlled trials to be included, after which he got 2232 results. From these, he did a title search in which 20 papers were shortlisted for abstract review. Thirteen were shortlisted from the abstract review. Out of these, three were shortlisted for this study.

Data Extraction and Management

A Microsoft Excel (Microsoft Corp., Redmond, WA, USA) spreadsheet was used for data extraction, following Cochrane's data collection form for intervention reviews. A pilot test was run by extracting data from random articles, and necessary adjustments were made accordingly.

Methodological Quality and Risk of Bias Assessment

The Newcastle-Ottawa scale [6] was employed to assess bias in nonrandomized studies across three domains: selection, comparability, and exposure. This scale employs a star scoring system, with a maximum total score of nine stars for each study (Table 1). 

**Table 1:** Newcastle-Ottawa scale assessing all observational studies for domains of selection, comparability, and outcome

Study	Selection	Comparability	Outcome	Overall quality	Overall score
Morasiewicz et al. [1]	****	*	***	Good	8 stars
Kim et al. [3]	***	*	***	Good	7 stars
Townshend et al. [7]	****	**	***	Excellent	9 stars

Results

Due to the heterogeneity between these three studies being too high, we could not perform a statistical analysis on them. Therefore, we discuss the results more descriptively. We looked into the four outcomes from the studies: the visual analogue score (VAS), length of hospital stay, complications, and the various test scores that were used to assess the function of the patients postoperatively (Table 2).

**Table 2:** Key findings of the studies included in the review VAS: Visual analogue scale, LOS: Length of stay, FAAM: Foot and ankle ability measure, AOS: Ankle osteoarthritis scale, SF-36 PCS: Short form-36 physical component score, AOFAS: American Orthopaedic Foot and Ankle Society

Author	Study design	Total participants (N)	VAS score	LOS (days)	Complications	FAAM score (0-100)	AOS at two years (0-100)	SF-36 PCS (mean improvement at one year)	AOFAS
Morasiewicz et al. [1]	Retrospective cohort	Illizarov: 21; Internal stabilization: 26	Illizarov: Pre-surgery 4.69, Post surgery 1.5 p = 0.037; Internal stabilization: Pre-surgery 4.71, post surgery 2.9, p = 0.044	Illizarov 5.29; Internal stabilization 5.71, p = 0.517	Illizarov 0.62; Internal stabilization 0.58, p = 0.066	Illizarov 79.38; Internal stabilization 70.11, p = 0.458	NR	NR	NR
Kim et al [3]	Retrospective cohort	Anterior 38; Transfibular 22	Anterior: Preoperative 5.18, postoperative 1.46; Transfibular: Preoperative 4.87, postoperative 1.27; p = 0.436	NR	Anterior 4; Transfibular 1; No comment on p-value	NR	NR	NR	Open: Preoperative 39.8, postoperative 58.3; Transfibular: Preoperative 44.5, postoperative 60.7, p = 0.274
Townshend et al [7]	Observational case series	Open 30; Arthroscopic 30	NR	Open 3.7; Arthroscopic 2.5, p = 0.05	NR	NR	Open 29.2; Arthroscopic 17.2, p = 0.05	Open 6.32; Arthroscopic 12.92, p = 0.01	NR

The VAS score was documented by Morasiewicz et al. and Kim et al. [1,3]. Morasiewicz et al. [1] compared Illizarov with internal stability using cannulated screws. In their study, it was found that the mean VAS score improved from 4.69 to 1.5 in Illizarov (p = 0.037) and from 4.71 to 2.9 in internal stability (p = 0.044). The improvement from preoperative to postoperative in both these techniques was statistically significant, but there was no statistical significance when comparing these scores between these techniques [1]. Kim et al. compared the anterior approach vs. transfibular approach, both using cannulated screws. The VAS score in the anterior approach improved from 5.18 preoperatively to 1.46 postoperatively, while it improved from 4.87 to 1.27 for the transfibular approach. Again, there was no statistical significance between these two techniques (p = 0.436) [3].

The length of hospital stay was calculated by Morasiewicz et al. [1] and Townshend et al. [7]. The latter compared open ankle arthrodesis vs. arthroscopic arthrodesis. The open ankle arthrodesis was done using the transfibular approach and used cannulated screws. The mean length of stay was 3.7 in the open technique while it was 2.5 in the arthroscopic technique, which was significantly less (p = 0.05) [7]. In the former study, the mean length of stay was 5.29 in the Illizarov technique vs. 5.71 in internal stability, which was not statistically significant (p = 0.517) [1].

The number of complications was compared in studies conducted by Morasiewicz et al. [1] and Kim et al. [3]. In the former study, the Ilizarov group reported a total of 13 complications, averaging 0.62 complications per patient (range 0-2). In the internal stability group, there were 15 complications, corresponding to an average of 0.58 complications per patient (range 0-2). This difference between the two means was not statistically significant (p = 0.066) [1]. In the latter study, it was found that four out of 38 patients in the anterior approach group had a complication, while only one patient out of 22 had a complication in the transfibular approach [3]. The author did not report whether this was statistically significant.

All three studies [1,3,7] used different scores to assess preoperative and postoperative functional improvement (Table 2). Morasiewicz et al. [1] used the functional outcome in the foot and ankle ability measure (FAAM) scale (0-100). For the group treated with the Illizarov technique, the mean FAAM score was 79.38, while it was 70.11 in the group treated with the internal stability technique. Although the score was higher in the Illizarov group, it was not statistically significant (p = 0.458) [1].

Kim et al. [3] used the American Orthopedic Foot and Ankle Society (AOFAS) score preoperatively and postoperatively. In the anterior approach, the mean AOFAS score was improved from 39.8 preoperatively to 58.3 postoperatively. While in the transfibular approach, it improved from 44.5 preoperatively to 60.7 postoperatively. The difference between these two groups was not statistically significant (p = 0.274) [3].

Townshend et al. [7] used the ankle osteoarthritis scale (AOS) score and the short form-36 (SF-36) health survey. The AOS score is scored from 0-100; however, in this case, the lower the score, the better. When comparing open vs. arthroscopic techniques, the AOS for the arthroscopic technique at two years was 17.2 on average, while it was 29.2 for the open technique. This was a statistically significant difference (p = 0.05) in favour of the arthroscopic technique [3].

The SF-36 has two components, namely the physical component score (PCS) and mental component score (MCS). The SF-36 PCS scores did not show a statistical difference at two years (mean improvement 8.12 in the open group vs. 11.45 in the arthroscopic group; p = 0.26). However, there was a significant difference between the groups at one year (mean improvement 6.32 in the open arthrodesis group vs. 12.92 in the arthroscopic arthrodesis group; p = 0.01) [3]. The SF-36 MCS scores for both techniques did not show statistical significance at one year (p = 0.68) or two years (p = 0.70) [3].

Kim et al. also measured the union rates and degree of hindfoot alignment. It was found that at the final follow-up, the average hindfoot alignment was 0.7° varus in the anterior approach group and 2.4° valgus in the transfibular approach group. The difference in postoperative alignment between the two groups was statistically significant (p = 0.05). The union rate of the anterior approach was 89.5% vs. 95.5% with the transfibular approach. This was not statistically significant (p = 0.400) [3].

Discussion

There are very few studies being conducted that look at different approaches and techniques being used in ankle arthrodesis. Although ankle arthrodesis is becoming more common than before, there is little consensus over which technique is better than the other. Each year in the United Kingdom, it's estimated that over 29,000 patients with symptomatic ankle osteoarthritis are referred to specialist foot and ankle surgeons within the National Health Service, with approximately 4,000 of these patients undergoing surgery [8].

**Figure 2:**
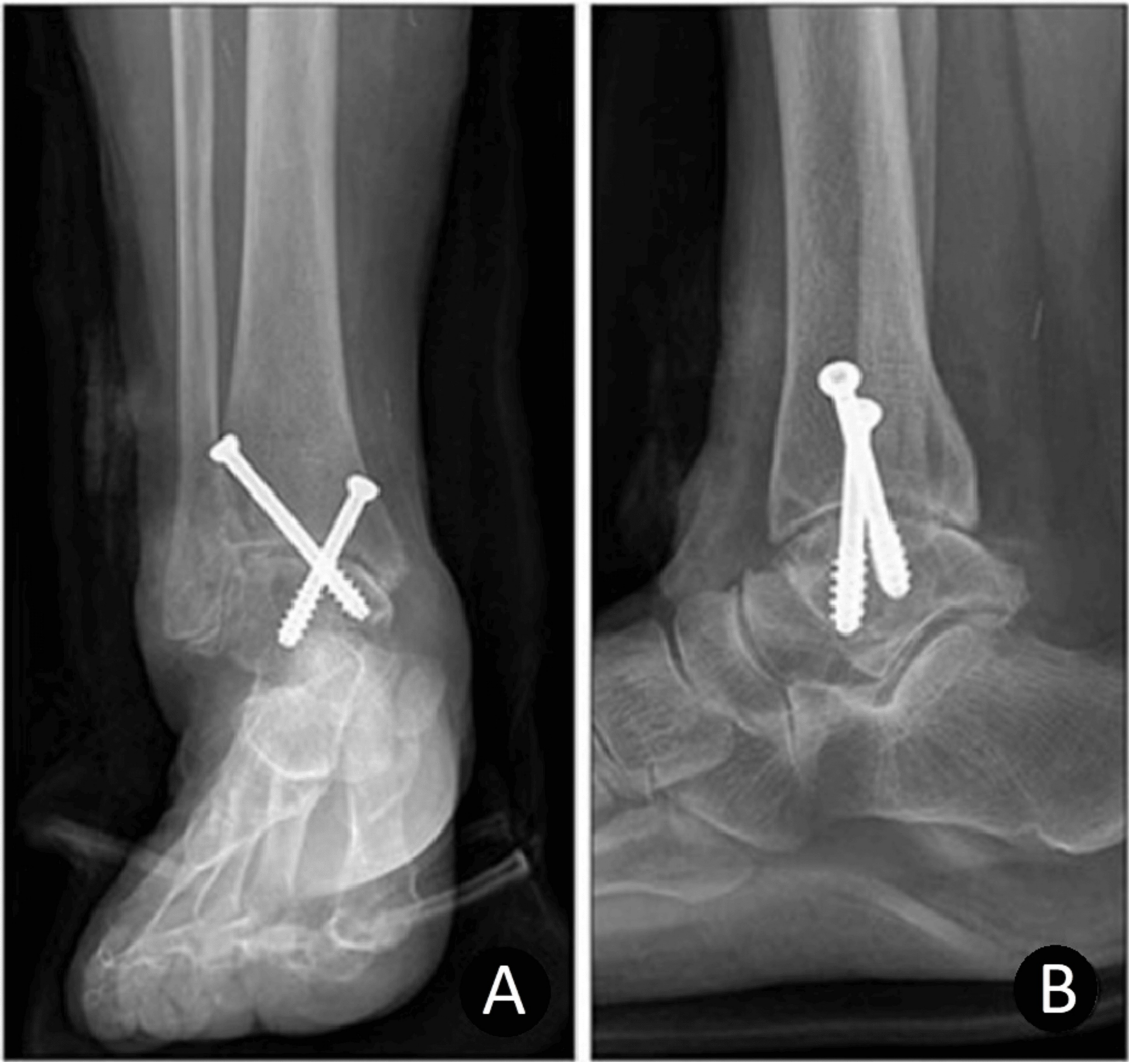
Ankle arthrodesis performed with the anterior approach using two 7.0 mm cannulated screws A: Anteroposterior view, B: Lateral view Copyright/license: This figure has been sourced from Kim et al. [3], which is an open-access article distributed under the terms of the Creative Commons Attribution Non-Commercial License (http://creativecommons.org/licenses/by-nc/4.0).

In our study, we looked at the anterior vs. transfibular approach, Illizarov vs. internal stability using the anterior approach, and open vs. arthroscopic approach. All of these techniques improved the patient’s function and pain postoperatively. In the anterior approach, one advantage is that it provides excellent visualization of the surgical area [9]. It also preserves the fibula and thus does not compromise future conversion to arthroplasty if required [4]. On the other hand, it puts the superficial peroneal nerve and anterior neurovascular bundle at greater risk (Figure 2) [10].

**Figure 3:**
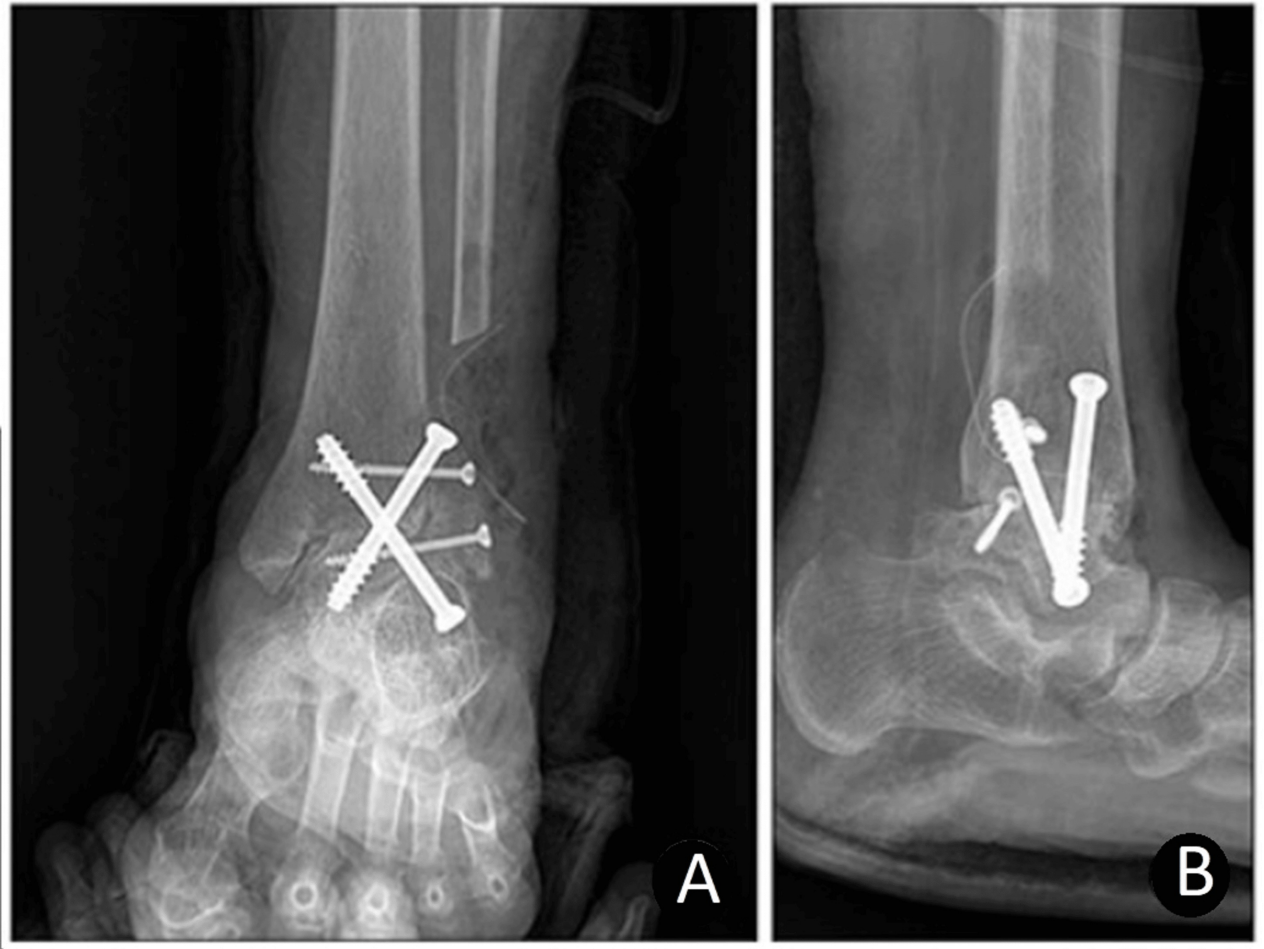
Ankle arthrodesis was performed using the transfibular approach with two 7.0 mm cannulated screws and fibular onlay bone graft A: Anteroposterior view, B: Lateral view Copyright/license: This figure has been sourced from Kim et al. [3], which is an open-access article distributed under the terms of the Creative Commons Attribution Non-Commercial License (http://creativecommons.org/licenses/by-nc/4.0).

In the transfibular approach, complications like wound infection, dehiscence, and delayed healing are less frequent compared to the anterior approach because the soft tissue on the lateral side is thicker. Additionally, the transfibular approach is beneficial for correcting deformities, as it provides access to the subtalar joint, sinus tarsi, and ankle joint. This approach not only allows for easier correction of issues like ankylosis but also minimizes shortening, maximizes contact area, and ensures greater stability [3]. With this approach, it is also possible to use the distal fibula as a bone graft or onlay graft, which is thought to provide more stability (Figure 3) [11].

Even with these techniques being significantly different from one another, the study by Kim et al. did not show any significant difference in the outcomes between anterior and transfibular techniques when it came to VAS scores, union rates, and AOFAS scores [3]. The one statistically significant outcome was the hindfoot alignment, which was 2.4 valgus on average in the transfibular approach compared to being slightly in varus in the anterior approach. In a previous study by Nihal et al., the ideal alignment after ankle arthrodesis was suggested to be the neutral ankle position, 0° to 5° of valgus angulation, with a slight external rotation. Therefore, the valgus alignment of the transfibular approach group compared to the anterior approach group may indicate better clinical results [3].

Morasiewicz et al. looked into Ilizarov vs. internal stability using cannulated screws. Ankle arthrodesis using the Ilizarov apparatus is especially helpful for patients with poor skin and soft tissue conditions, significant or complex deformities, limb shortening, or weak bone quality. It also has a higher success rate in achieving bone union [1]. All the outcomes the author looked into were not statistically significant to prove that either of these techniques was superior to the other, even though the mean VAS score, length of stay, and FAAM scores were better in Ilizarov than internal stability. Also, there were no reports of non-union in the Ilizarov group, while there were four cases of non-union in the internal stability group, which were then treated with Ilizarov fixation [1].

Townshend et al. compared open vs. arthroscopic techniques for ankle arthrodesis. It was found that both the open and arthroscopic approaches showed good clinical outcomes after the one and two-year follow-ups. However, the arthroscopic approach was significantly better when it came to the length of hospital stay, AOS scores, and SF-36 PCS score at the one-year follow-up [7]. This study showed that the arthroscopic approach was superior to the open approach.

Limitations

This review has several limitations. First, it included only studies published in English, which may have led to the exclusion of relevant research conducted in non-English-speaking countries, potentially introducing language bias. Second, studies published more than five years ago were excluded to focus on recent advancements and techniques in the field; however, this restriction may have omitted earlier but still relevant evidence. Lastly, there was notable heterogeneity among the included studies in terms of design, patient populations, interventions, outcome measures, and follow-up durations. This variability limited the ability to perform a robust meta-analysis and may affect the generalizability of the findings.

## Conclusions

It can be concluded that all six different techniques reviewed here improve the clinical outcomes of patients. However, the arthroscopic technique shows the most promising and effective outcomes when it comes to length of hospital stay, AOS, and SF-36 scores. Further research needs to be conducted in this area to help surgeons arrive at a consensus on which technique to use and improve on. Also, there should be a consensus as to which functional scores to use to enable ease in the comparison of techniques and calculate the outcomes of patients.
